# Dyslipidemia among HIV-infected patients in Ethiopia: a systematic review and meta-analysis

**DOI:** 10.1186/s12879-023-08910-9

**Published:** 2024-01-02

**Authors:** Ousman Mohammed, Ermiyas Alemayehu, Habtu Debash, Melaku Ashagrie Belete, Alemu Gedefie, Mihret Tilahun, Hussen Ebrahim, Daniel Gebretsadik Weldehanna

**Affiliations:** Department of Medical Laboratory Sciences, College of Medicine and Health Sciences, Wollo University, Dessie, Ethiopia

**Keywords:** Dyslipidemia, Serum lipid abnormality, Antiretroviral therapy, HIV/AIDS, Ethiopia

## Abstract

**Background:**

Dyslipidemia is responsible for more than half of the global ischemic heart disease (IHD) and more than 4 million deaths annually. Assessing the prevalence of dyslipidemia can be crucial in predicting the future disease development and possible intervention strategies. Therefore, this systematic review and meta-analysis was aimed at assessing the pooled prevalence of dyslipidemia in HIV-infected patients.

**Methods:**

Electronic databases such as EMBASE, Google Scholar, PubMed, Web of Science, ResearchGate, Cochrane Library, and Science Direct were searched for articles and grey literature. All relevant studies found until our search period of May 24, 2023 were included. The Newcastle–Ottawa Quality Assessment Scale was used to assess the quality of the included studies. The data were extracted in Microsoft Excel. The STATA version 14 software was used to conduct the meta-analysis. I^2^ and Cochran’s Q test were employed to assess the presence of heterogeneity between studies. Due to the presence of heterogeneity, a random effect model was used. The publication bias was assessed using the symmetry of the funnel plot and Egger's test statistics. Moreover, subgroup analysis, and sensitivity analysis were also done.

**Results:**

A total of nine studies that reported the prevalence of dyslipidemia were included. The overall pooled prevalence of dyslipidemia among HIV-infected patients in Ethiopia was 67.32% (95% CI = 61.68%–72.96%). Furthermore, the overall pooled estimates of dyslipidemia among ART-taking and treatment-naïve HIV-infected patients were 69.74% (95% CI: 63.68–75.8, I^2^ = 87.2) and 61.46% (95% CI: 45.40–77.52, I^2^ = 90.3), respectively. Based on lipid profile fractionations, the pooled estimates for high total cholesterol (TC) were 39.08% (95% CI: 31.16–46.99), high triglycerides were 38.73% (95% CI: 28.58–48.88), high low density lipoprotein (LDL-c) was 28.40% (95% CI: 17.24–39.56), and low high density lipoprotein (HDL-c) was 39.42% (95% CI: 30.47–48.38).

**Conclusion:**

More than two-thirds of HIV-infected patients experienced dyslipidemia. Therefore, it's critical to regularly evaluate lipid alterations in HIV-infected patients in order to prevent the onset of atherosclerosis and other cardiovascular problems.

**Supplementary Information:**

The online version contains supplementary material available at 10.1186/s12879-023-08910-9.

## Introduction

Dyslipidemia is an imbalance of blood lipids associated with elevated concentrations of low-density lipoprotein cholesterol (LDL-c), total cholesterol (TC), triglycerides (TG), and low concentrations of high-density lipoprotein cholesterol (HDL-c) [1–3]. It is responsible for more than half of global ischemic heart disease (IHD) and more than 4 million deaths annually [4]. Mechanisms for the development of dyslipidemia are still unclear and are proposed to be multifactorial in HIV patients [5]. Even after controlling for conventional cardiovascular disease (CVD) risk factors, human immunodeficiency virus (HIV)-infected people still have a higher risk of developing CVD, including acute myocardial infarction (MI). The majority of HIV-positive patients' fatalities result from cardiovascular problems, liver disease, and renal failure, all of which have links to the virus, the host, and antiretroviral therapy (ART) variables [6].

Cardiovascular diseases (CVDs) are two times more likely to occur in people with dyslipidemia than in people with normal lipid levels [7]. Africa is witnessing significant shifts in population health, characterised by an increasing prevalence of CVDs, which are expected to surpass infectious diseases as the primary cause of death by 2030 [8]. The overall pooled prevalence of dyslipidemia in Africa's general population was 52.8% [9]. The prevalence of dyslipidemia among HIV-infected patients in Africa, on the other hand, ranged from 13 to 70% [10]. The prevalence of Dyslipidemia in the African population was 25.5% for high total cholesterol concentrations, 37.4% for low HDL cholesterol concentrations, 28.6% for elevated LDL cholesterol concentrations, and 17.0% for elevated triglyceride concentrations. Dyslipidemia is exacerbated by those living with HIV and other chronic conditions [11].

ART that is started on time has been shown to significantly slow down the HIV virus from multiplying and destroying CD4 cells and then lengthen life in HIV-infected individuals [12–14]. Despite the fact that HIV patients on ART have an increased life expectancy, degenerative diseases induced by HIV, ART, or inflammation are also taken into account [15–19]. These diseases include Dyslipidemia, atherosclerosis, and insulin resistance. Moreover, increased exposure to ART might be associated with increased CVD diseases [20, 21].

HIV patients should undergo lipid profile testing when initiating treatment or making changes to ART. Following that, if their previous lipid test results were normal, they should have their lipid profiles done annually, or every six months if they were abnormal [22]. Determining the prevalence of dyslipidemia can be critical for predicting future disease development. In Ethiopia, even though there were few studies conducted to determine the prevalence of dyslipidemia among HIV-infected patients, the pooled prevalence is not yet known [23]. Moreover, the studies were conducted in single-study settings with a small sample size. Therefore, providing the pooled prevalence of dyslipidemia among HIV-infected patients might be more informative and crucial for concerned bodies to make decisions on the management and monitoring of disease progress to prevent further cardiovascular complications.

## Methods

### Protocol registration

The Preferred Reporting Items for Systematic Review and Meta-Analyses (PRISMA) statement (Supplemental Table 1) was followed for this review [24]. The study protocol has been uploaded to the International Prospective Register of Systematic Reviews (PROSPERO) with the registration number (CRD42023420768).

### Search strategy

Systematic electronic searches using databases such as EMBASE, Google Scholar, PubMed, Web of Science, ResearchGate, Cochrane Library, and Science Direct were done from January to May 24, 2023, to retrieve all relevant primary articles reporting the prevalence of dyslipidemia among HIV-infected patients in Ethiopia. Moreover, other sources such as journal homepages, institutional repositories, and bibliographies were searched to retrieve eligible studies. With the aid of an expert on the review topic field, search strategies are created that incorporate free-text phrases and any relevant subject indexing (such as MeSH) to anticipate returning acceptable papers. Boolean logic was used to combine the following keywords to create the search protocol: "dyslipidemia" OR “atherogenic dyslipidemia" OR "lipid profile alteration" OR "biochemical derangement" OR "lipid profile abnormalities" OR "lipid profile elevation" AND "HIV/AIDS" and each Ethiopian region. The search results were managed using the EndNote X7 software. To identify potentially suitable papers, the two reviewers (OM and EA) blindly examined the titles, abstracts, and full-text search results. Likewise, the whole text of selected papers was thoroughly reviewed in light of the inclusion criteria. For duplicate studies, the first version or the one with all the necessary data was used. Any disagreements that occurred during screening were settled by consensus.

### Eligibility criteria

#### Inclusion criteria

This meta-analysis and systematic review comprised observational studies carried out in Ethiopia among adults with HIV who were older than 18 years. The inclusion criteria included full-length studies reporting the prevalence of dyslipidemia and/or having the ability to determine the prevalence of dyslipidemia among HIV-infected patients. All relevant studies found until our search period of May 24, 2023, were included. However, no restrictions were applied regarding region or gender. Furthermore, grey literature written in the English language was also included.

### Exclusion criteria

Exclusion criteria included studies that either failed to describe the prevalence of dyslipidemia or lacked relevant data to calculate it. Other exclusion criteria include studies that are duplicate, unavailable full texts, abstract-only papers containing no extracted data or information, clinical trials, case reports, case series studies, letters to the editor, conference proceedings, or review articles.

#### Outcome measurement

The outcome variable in this study was prevalence of dyslipidemia in HIV-infected individuals, which was defined using National Cholesterol Education Programme (NCEP) [25].

#### Definition

Dyslipidemia: either TC concentrations ≥ 200 mg/dL or TG of ≥ 150 mg/dL, or LDL-c > 130 mg/dL, or HDL-c < 40 mg/dL for men and < 50 mg/dL for women [25].

#### Data extraction

Two freelance authors (OM and EA) extracted the data from each study and entered it in a customised way into a Microsoft Excel sheet. Information was gathered about the authors, the publication year, the sample size, the study design, the region, the status of ART, and the study settings. Moreover, data on lipid profiles (TG of ≥ 150 mg/dL, LDL-c > 130 mg/dL, or HDL-c < 40 mg/dL for men and < 50 mg/dL for women) were also extracted. These authors then compared the outcomes and addressed discrepancies through consensus-based talks after using standardised data extraction forms to obtain data from the entire text of potentially eligible studies. When studies lacked sufficient methodological information or the substance was unclear, the principal authors were approached for clarification via an official email address or phone number. Any disagreements between two independent authors were settled by the third author.

#### Quality assessment

Newcastle–Ottawa the methodological quality and bias risk of the included studies were evaluated using the scale designed for cross-sectional study quality evaluation [26]. All eligible studies were reviewed, and only those of good quality or above were included in the final analysis (Supplemental Table 2 and 3). Two authors (OM and EA) independently assessed the quality of each featured work. Before determining the final evaluation score, all authors who participated in data extraction appraised the quality of the extracted studies.

#### Statistical analysis

STATA-14 was used to analyse the retrieved data. Because there was significant variability, the pooled prevalence of dyslipidemia was calculated using a random effect model. The heterogeneity of the included studies was evaluated using the forest plot, Cochran's Q (2 test), the I^2^ test, and the *p*-value. In a pooled study, an I^2^ statistic value of less than 25% was deemed to have no heterogeneity, 25 to 50% was deemed to have low heterogeneity, more than 50% was deemed to have moderate heterogeneity, and 75% was deemed to have high heterogeneity [27, 28]. To depict the pooled prevalence and 95% CI, forest plots were used. Subgroup analysis was done based on study setting, year, design, and ART status to show trends and related issues over time. The influence of one study on the combined estimates from the other studies was also examined using leave-one-out sensitivity analysis [29]. The funnel plot's symmetry was visually inspected, and Egger's test statistics were used to assess publication bias among the studies [30]. The presence of publication bias was declared with a *p*-value less than 0.05.

## Result

### Search results

**Fig. 1:**
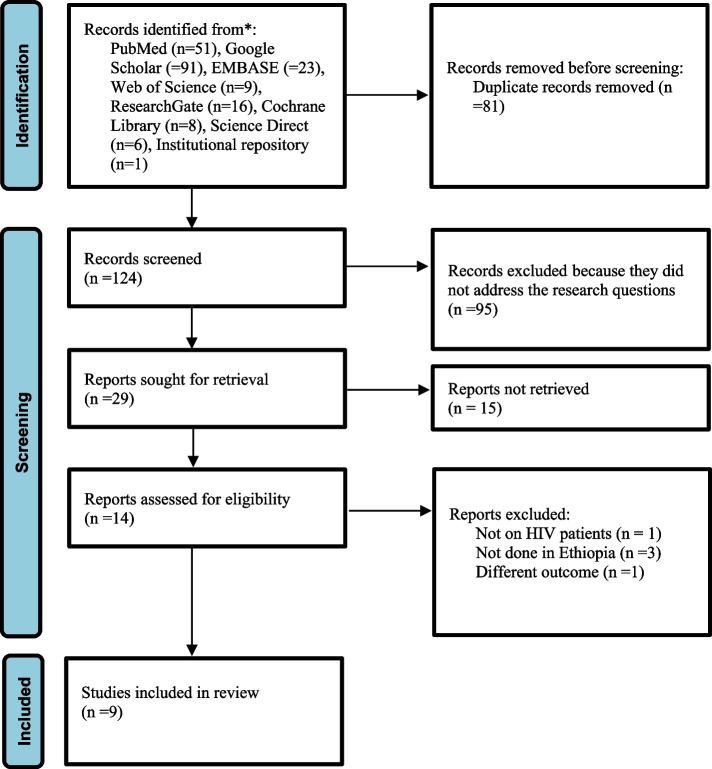
Flow chart of studies’ search and retrieval process

Figure 1 depicts the flow chart and selection technique for determining the pooled prevalence of dyslipidemia among HIV/AIDS patients. Through electronic searches, a total of 205 articles were discovered, and 124 non-duplicate articles were reviewed. Approximately 81 duplicate articles were removed, and another 101 studies were discarded because they did not respond to the research questions. The remaining 14 studies were excluded for various reasons after 23 full-text papers were evaluated for eligibility. As a result, only nine studies that reported the prevalence of dyslipidemia were included. Furthermore, all of the included studies were of good quality, according to the Newcastle–Ottawa Quality Assessment Form (Supplemental Table 2 and 3).

### Overview of included studies

In the present study, nine original articles published until May 24, 2023, consisting of 2274 study participants [23, 31–38], were included. All the included studies were conducted in Ethiopia's two regions and one city administration. More than half of the studies were conducted in Addis Ababa [22, 32–35], and only one study was found in the southern [31] part of Ethiopia. The earliest study [31] was published in 2012, and the most current study [23] was published in 2023. The included studies' sample sizes ranged from 63 to 407 individuals. All the included studies were conducted at the health institution level, and no community-based study was found. The mean age of the study participants varied from 33.9 to 44.2 years. The higher prevalence of dyslipidemia (82.3%) was seen among ART-taking HIV-infected patients and the lower (53.5%) among treatment-naïve groups. Based on evidence from three studies [31, 34, 35], more than one-third of the HIV-infected patients do have a TC/HDL-c ratio > 5. Furthermore, Tadewos et al. [31] found the highest (79.6%) prevalence of dyslipidemia in at least the lipid profile, while Assefa et al. [23] reported the lowest (55.2%) prevalence (Table 1).

**Table 1:** Overview of included studies conducted in Ethiopia (*N* = 2274), 2023

Authors (Year)	Region	Study design	Mean age (year)	Sample size	Dyslipidemia in at least one lipid profile N (%)	Dyslipidemia on ART N (%)	Dyslipidemia on treatment naïve N (%)	TC/HDL-c ratio > 5 (%)
Tadewos et al. (2012) [31]	Southern	Cross-sectional	37.2 ± 8.7	226	180 (79.6)	93 (82.3)	87 (76.9)	43.4
Kemal et al. (2020) [32]	Addis Ababa	Cross-sectional	44.2 ± 9.016	353	264 (74.8)	264 (74.8)	NA	NA
Aklog A. (2019) [33]	Addis Ababa	Cross-sectional	33.9 ± 9.7	89	62 (69.7)	62 (69.7)	NA	NA
Wondiferaw et al. (2014) [34]	Addis Ababa	Cross-sectional	35.31 ± 7.20	228	145 (63.6)	84 (73.7)	61 (53.5)	32.5
Simeneh T. (2020) [35]	Addis Ababa	Cohort	39.7 ± 10	63	49 (77.8)	49 (77.8)	NA	30.2
Tilahun et al. (2022) [36]	Amhara	Cross-sectional	35.31 ± 7.20	228	145 (63.6)	84 (73.7)	61 (53.5)	NA
Gebrie et al. (2020) [37]	Amhara	Cross-sectional	38.6 ± 10.3	407	260 (63.9)	260 (63.9)	NA	NA
Fiseha et al. (2021) [38]	Amhara	Cross-sectional	41.2 ± 14.4	392	235 (59.9)	235 (59.9)	NA	NA
Assefa et al. [23]	Addis Ababa	Cross-sectional	43.5 ± 11.27	288	159 (55.2)	159 (55.2)	NA	NA

*NA* Not available, *TC/HDL-c* total cholesterol/high density lipoprotein ratio

### Prevalence of dyslipidemia among HIV-infected patients

**Fig. 2:**
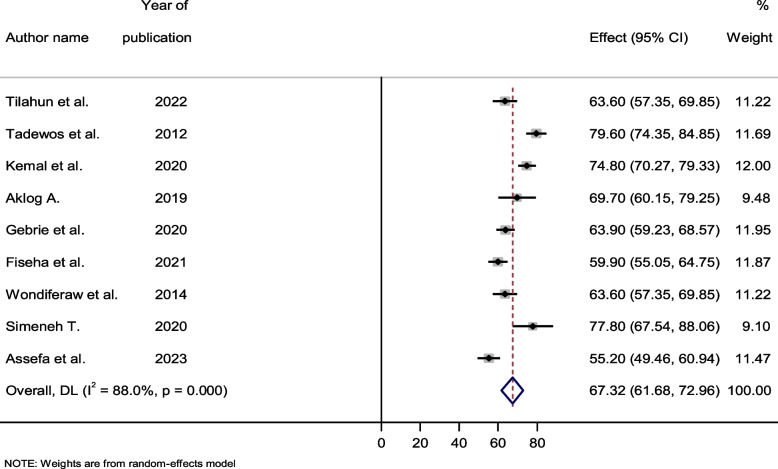
Forest plot showing the pooled prevalence of dyslipidemia among HIV- infected patients, 2023

According to the current meta-analysis and systematic review, the overall pooled prevalence of dyslipidemia among HIV-infected individuals in Ethiopia was 67.32% (95% confidence interval (CI): 61.68–72.96%). With a Q test (Tau-squared) value of 66.47 (degree of freedom, d.f. = 8, *p*-value < 0.001), high level of heterogeneity was discovered, and I^2^ was judged to be 88.0% for the degree of inconsistency (Fig. 2).

### Pooled prevalence of lipid profile alteration among HIV-infected patients in Ethiopia

**Fig. 3:**
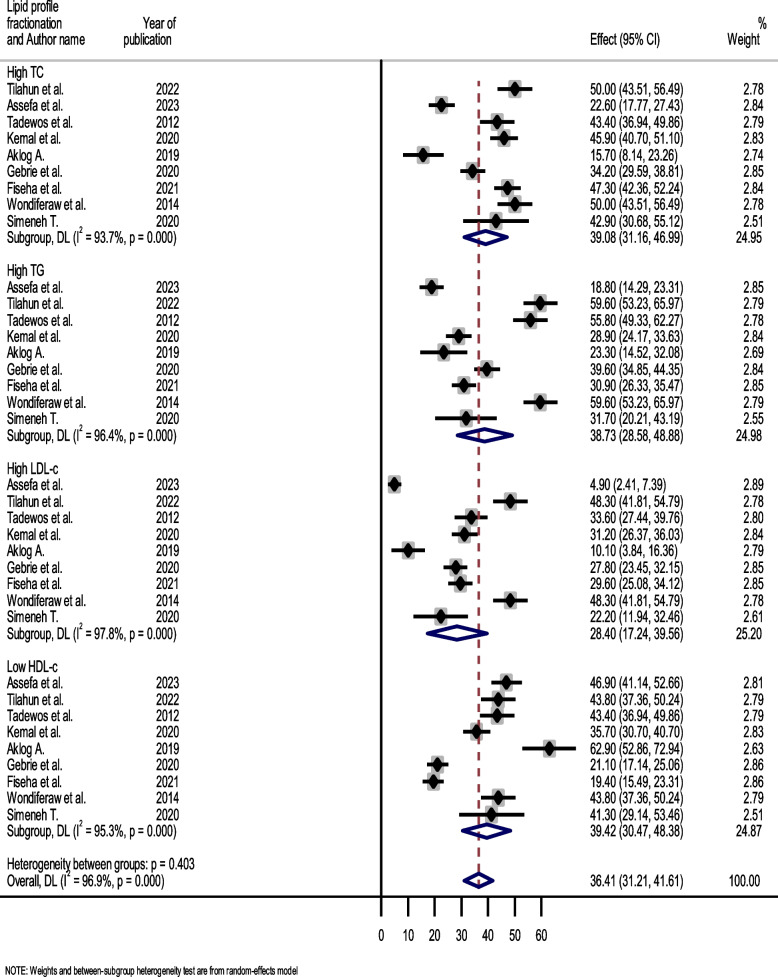
Forest plot showing the pooled prevalence of lipid profile alterations among HIV-infected patients, 2023

The forest plot analysis by lipid profile showed that there was marked variation across the lipid profile fractionations. The most frequent lipid profile abnormalities were seen in serum total cholesterol and triglycerides. The pooled point estimates for high total cholesterol (TC) were 39.08% (95% CI: 31.16–46.99; I^2^ = 93.7, *p* < 0.001), high triglycerides (TG) were 38.73% (95% CI: 28.58–48.88; I^2^ = 96.4, *p* < 0.001), high low density lipoprotein (LDL-c) was 28.40% (95% CI: 17.24–39.56; I^2^ = 97.8, *p* < 0.001), and low high density lipoprotein (HDL-c) was 39.42% (95% CI: 30.47–48.38; I^2^ = 95.3, *p* < 0.001) (Fig. 3).

### Subgroup analysis by study year and design

**Fig. 4:**
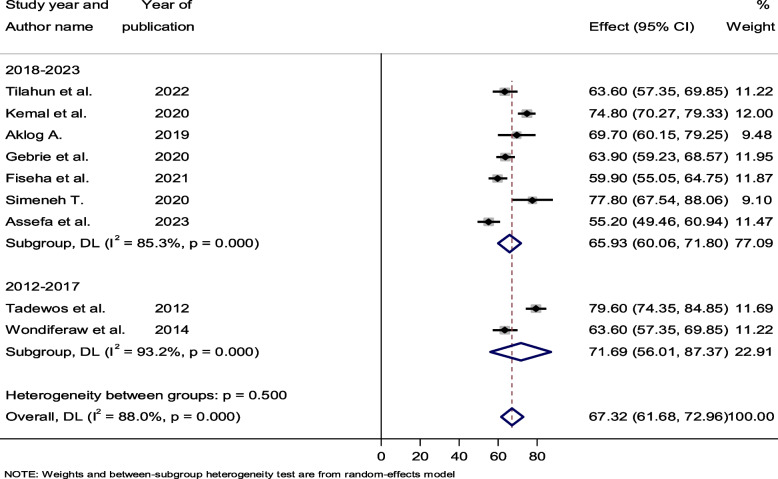
Forest plot showing the pooled prevalence of dyslipidemia among HIV-infected patients by study year, 2023

**Fig. 5:**
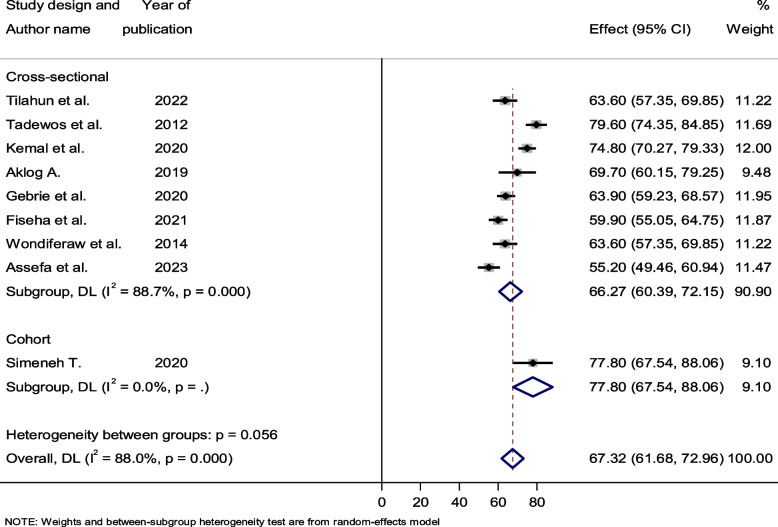
Forest plot showing the pooled prevalence of dyslipidemia among HIV-infected patients by study design, 2023

Subgroup analysis based on study setting, ART status, study design, and study year was considered to identify the most likely reason for heterogeneity. Based on the study year, we separated the included studies into two groups (2012–2017 and 2018–2023) to assess the prevalence of dyslipidemia over time. The pooled prevalence of dyslipidemia was determined to be 71.69% (95% CI: 56.01–87.37, I^2^ = 93.2) between 2012 and 2017, and 65.93% (95% CI: 60.06–71.80, I^2^ = 85.3) between 2018 and 2023. With a *p*-value of 0.50, there was no significant heterogeneity between groups (Fig. 4). In terms of study design, only one study used cohort study designs, while the other eight used cross-sectional study designs. In mong cross-sectional studies, the pooled prevalence of dyslipidemia was 66.27% (95% CI: 60.39–72.15, *p* < 0.001). In these cross-sectional studies, there was markedly high heterogeneity, with an I^2^ value of 88.7% (Fig. 5).

### Subgroup analysis by ART status

**Fig. 6:**
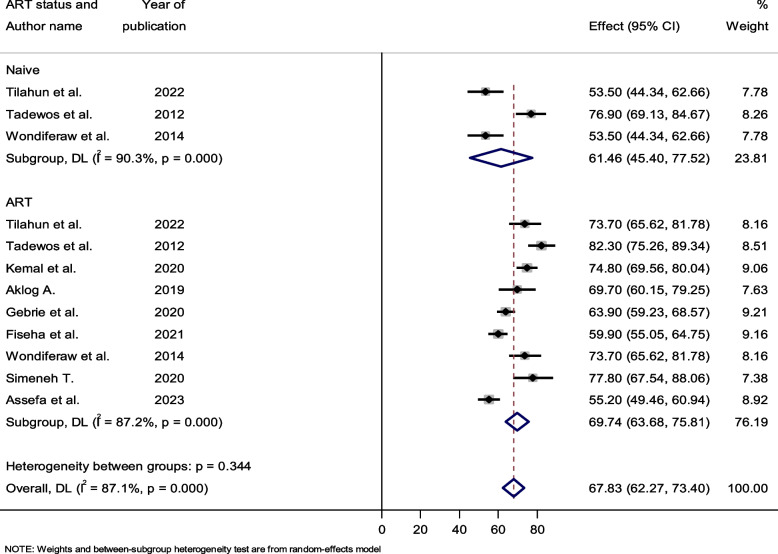
Forest plot showing the pooled prevalence of dyslipidemia among ART taking and naïve HIV-infected patients, 2023

Out of all the included studies, only three of them reported both the prevalence of dyslipidemia in groups that were not on ART and those that were. Furthermore, every other study was limited to HIV-infected patients receiving antiretroviral therapy. HIV patients on ART exhibited varying levels of dyslipidemia: 55.20% to 82.30% and 53.50% to 76.90%, respectively, compared to those who were not on ART treatment. Additionally, for HIV-infected individuals who were not on ART, the total pooled estimates of dyslipidemia were 61.46% (95% CI: 45.40–77.52; I2 = 90.3, *P*-value < 0.001) and 69.74% (95% CI: 63.68–75.8; I^2^ = 87.2, *P*-value < 0.001), respectively (Fig. 6).

### Subgroup analysis by study setting

**Fig. 7:**
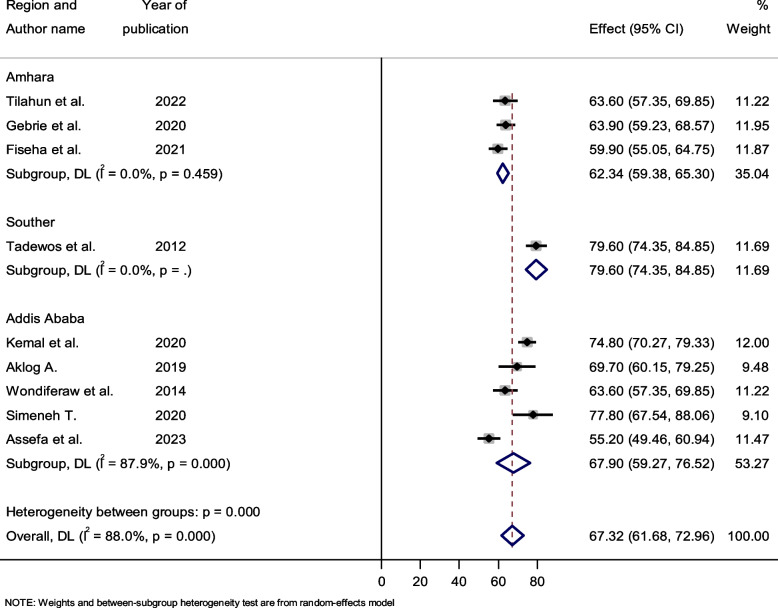
Forest plot showing the pooled prevalence of dyslipidemia among HIV-infected patients by study setting**,** 2023

The pooled prevalence of dyslipidemia among HIV-infected patients ranged from 62.34% (95% CI: 59.38–65.30) in the Amhara region to 79.60% (95% CI: 74.35–84.85) in the southern part of Ethiopia. The prevalence estimates between studies by sub-region revealed significant heterogeneity in Addis Ababa (heterogeneity, *p* < 0.0001), but no heterogeneity in the Amhara region (Fig. 7).

### Publication bias

**Fig. 8:**
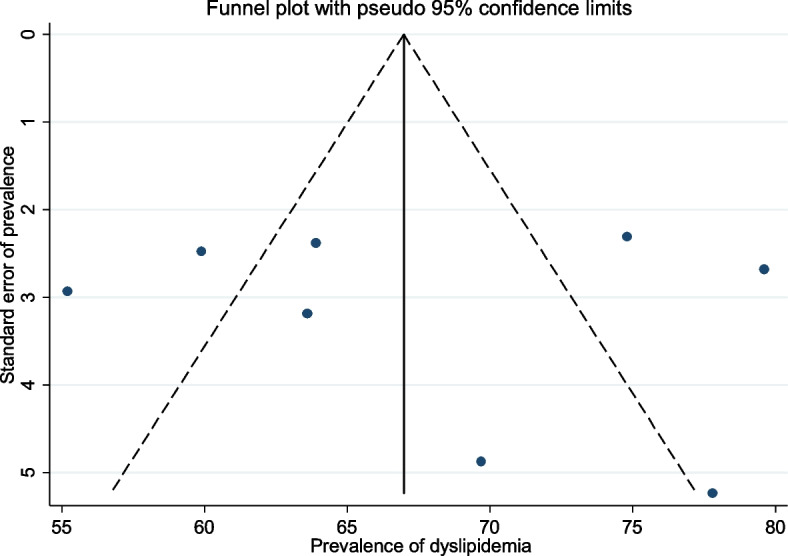
A publication bias assessment plot of the reported prevalence of atherogenic dyslipidemia among HIV-infected patients across Ethiopian studies, 2023

Publication bias was assessed using funnel plots and Egger at a 5% significant level. There was no statistical evidence of publication bias in the pooled estimates of dyslipidemia. The Egger test was non-significant (*p* = 0.85), and the funnel plot was nearly symmetric (Fig. 8).

### Sensitivity analysis

**Fig. 9:**
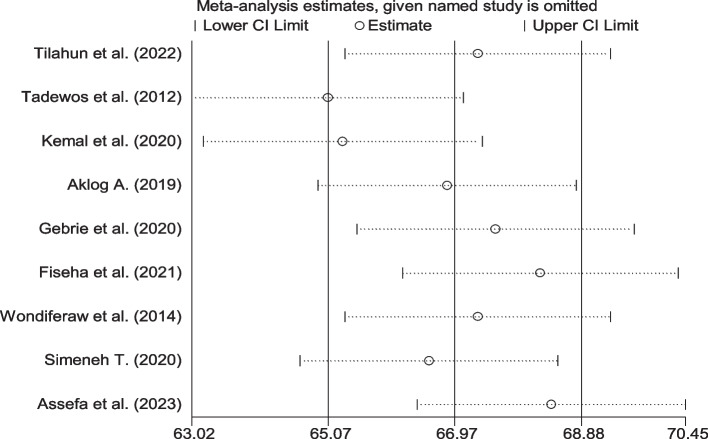
Sensitivity analysis for single study influence of pooled estimate, 2023

A sensitivity analysis was applied to assess the effect of a single study on the total effect size. The sensitivity analysis showed that no single study had an effect on the overall prevalence of dyslipidemia among HIV-infected patients (Fig. 9).

## Discussion

Dyslipidemia has emerged as a major risk factor for cardiovascular disease in African countries [39]. It is associated with ART, HIV, or both and is known to increase the risk of cardiovascular disease [40, 41]. This review study was the first to characterise the pooled prevalence of dyslipidemia among HIV-infected patients because no previous study has looked at the countrywide pooled prevalence of dyslipidemia among people with HIV. According to our findings, the pooled prevalence of dyslipidemia among HIV-infected individuals across the country in Ethiopia was 67.32% (95% CI = 61.68%–72.96%), which is comparable to a study in Brazil that found a prevalence of 66.7% [42] and 73.3% in Nigeria [43]. Not unexpectedly, we found that among HIV patients, dyslipidemia was highly prevalent. Due to changes in blood lipid levels, HIV and ART have been linked to an increased risk of CVD [40, 41]. Therefore, ongoing dyslipidemia monitoring would be beneficial for HIV-positive individuals in order to take prompt preventive or remedial action.

The present pooled prevalence, however, is greater than previous studies in Zimbabwe (60%) [44], the general population (ranging from 23 to 25% [45], south-east Malawi (15.5% [46], and Uganda (6.4%) [47], the African pooled prevalence of 52.80% [9], Thailand 51% [48], India (50.7% [49], and China (32.2%) [50]. This increased prevalence of dyslipidemia among HIV-positive persons could be attributed to both ART and the virus itself. It can be caused by a variety of mechanisms, like viral particle competition on lipid metabolism receptors, such as cellular retinoic acid binding protein (CRABP-1) and LDL receptor-related protein (LRP), which reduces lipid clearance; suppression of lipoprotein lipase activity; increased liver beta-apolipoprotein levels; increased hepatic synthesis of very low-density lipoprotein (VLDL); increased cytokine levels (TNF and IL) [51, 52]. On the other hand, the current pooled prevalence is lower than in previous studies in Kenya (79.6%) [53] and South Africa (90.0%) [54]. Differences in the study population, sample size, methodology, level of urbanisation, cut-off values, lifestyle, and socioeconomic status may account for this discrepancy.

Antiretroviral therapy, on the other hand, is linked to an increase in the incidence of lipoatrophy, dyslipidemia, and irregularities of fat distribution in HIV patients. Studies found that HIV-infected patients receiving ART had a considerably greater incidence of dyslipidemia than the naive group and that this incidence rose sharply with cumulative ART exposure [55]. We found that more than two-thirds of ART-taking HIV-infected patients had experienced dyslipidemia. According to the evidence from the current review study, the prevalence of dyslipidemia was slightly higher among ART-taking HIV-infected patients. Similar to the current finding, a study in China found that the pooled prevalence of dyslipidemia among ART-taking patients was slightly higher than that among those without ART [56]. Additionally, a number of studies conducted in African nations found that individuals subjected to ART had a higher prevalence of dyslipidemia, ranging from 36.90 to 85% [57–60]. Another study in Malaysia found that 82.30% of 1,583 antiretroviral medication-taking HIV-infected patients had experienced dyslipidemia [61]. Furthermore, comprehensive evidence revealed that HIV-infected individuals receiving ART had greater levels of dyslipidemia than those who were not receiving treatment. The issue is complex and has been linked to both HIV infection itself and the use of antiretroviral medications [62–66]. The ART itself increases biosynthesis and reduces hepatic clearance of serum cholesterol, thereby leading to dyslipidemia [41, 42].

The pooled prevalence of elevated total cholesterol in the current review was 39.08%, comparable to studies in Iran at 41.6% [67], Poland at 37% [68], Ethiopian studies at 34.08% [69], Turkish studies at 37.50% [70], and Lebanon at 36.90% [71], but higher than China at 33% [72], Tanzania at 30.4% [73], and Korean studies at 6.00% [74]. However, studies in Saudi Arabia (54% [75]) and South Africa (67.30% [76]) found a greater prevalence of total cholesterol elevation. Furthermore, the pooled prevalence of increased LDL cholesterol concentrations in this review study was 28.40%, which is comparable to earlier studies in Iran at 35.5% [67], Lebanon at 32.1% [71], Poland at 31% [68], Switzerland at 20.80% [77], and China at 24.80% [72]. On the other hand, the present result of 28.40% elevated LDL-c is lower than other studies in Ethiopia (41.13% [69]) and Turkey (44.5%) [70]. Environmental factors associated with persistent HIV infection include nutrition, genetics, ART-induced dyslipidemia, and adipose tissue dysfunction. All of these factors are likely to contribute to metabolic illness [78, 79].

Regarding the low concentrations of HDL cholesterol, we found 39.42%, which is comparable to Lebanon's 32.10% [71]. In contrast, this is higher than south-east Malawi's 15.90% [46], Poland's 20.50% [68], Turkey's (21.10%) [70], Switzerland's 2.80% [77], China's 24.80% [72], Cameroon's 19.5% [80], and Botswana's (6.3%) [81]. Likewise, studies in Iraq (40.90% [82], Turkey (44.50%) [70], Uganda (85.60%) [47], Tanzania (43.60%) [83], Brazil (53.50%) [42], and Nepal (56.70% [84]) reported higher prevalence than our study. This disparity may be explained by the fact that the current study is a meta-analysis of nine studies, whereas the majority of the earlier studies were original studies conducted in single-study settings. Furthermore, the prevalence of aberrant TC/HDL ratios (> 5) ranged from 30.20% [35] to 43.40% [31], consistent with Eritrea's 33.20% [85] but higher than Southeast Malawi's 3.80% [46]. Because low HDL cholesterol is a component of HIV-induced dyslipidemia, the metabolism of HDL cholesterol in these people is also hindered. Although the exact causes of HIV infection and HAART-induced HDL cholesterol lowering are unknown, hypoalphalipoproteinemia is a common observation in HIV patients [86].

For the pooled prevalence of elevated triglycerides, we found 38.73%, which is similar to other Ethiopian studies of 39.70% [87], Ethiopian meta-analysis 48.15% [69], Eastern India 37.70% [88], and Iraq 41.60% [82]. However, higher than African studies (17.0% [73], Korean 32.10% [74], south-east Malawi 28.70% [46], Cameroon 7.8% [80], Uganda 29.60% [47], Switzerland 12.50% [77], while lower than studies in Poland 52% [68], India 93.80% [89], and Nepal 48.30% [84]. The wide variety of dyslipidemia seen in numerous studies, including the current review, may be explained by differences in study population, genetic factors, physical activity, dietary habits, consumption of alcohol, smoking, overweight or obesity, ART duration, and ART regimens among the studies. Notably, due to the complex and multidirectional relationships among diet, genetic factors, ART, viral replication, chronic inflammation, and lipid metabolism, careful monitoring and treatment of lipid levels are likely more informative in individuals with HIV infection than in those without the infection [90].

With respect to the patterns of dyslipidemia prevalence over time, the pooled estimations of dyslipidemia in studies conducted between 2012 and 2017 were 71.69%, while in studies conducted between 2018 and 2023, they were found to be 65.93%. Assefa et al. [23] in 2023 found the lowest prevalence of dyslipidemia (55.20%), whereas Tedewos et al. [31] in 2012 reported the highest prevalence (79.60%) of the condition. Nonetheless, when we look at the general trends in the prevalence of dyslipidemia among HIV-infected patients, we can observe that there was some fluctuation over the year (Fig. 4). The extensive search of the literature across all relevant databases, the careful screening of relevant studies, and the comprehensive evaluation of quality to eliminate quality bias are among the strong points of this review. However, due to inconsistencies in the data from the included studies, associated risk factors were not assessed.

## Conclusion

More than two-thirds of HIV-infected patients experienced dyslipidemia. Dyslipidemia screening for newly diagnosed HIV-infected individuals should be a crucial component of HIV management. Therefore, it's critical to regularly evaluate lipid alterations among HIV-infected patients in order to prevent the onset of atherosclerosis and other cardiovascular problems.

### Supplementary Information

**Additional file 1. **

**Additional file 2: Supplementary Table 2.** Detailed Newcastle-Ottawa Quality Assessment Form for Cohort Studies. **Supplementary Table 3.** Detailed Newcastle-Ottawa Quality Assessment Form for each cross-section Studies.

## Data Availability

All the datasets used and/or analyzed during the current study are available in the manuscript.
