# Supplementary material for: Dyslipidemia among HIV-infected patients in Ethiopia: a systematic review and meta-analysis
Source: BMC Infect Dis. 2024 Jan 2;24:27. doi: 10.1186/s12879-023-08910-9 (PMC10763320; doi:10.1186/s12879-023-08910-9)
Supplement: Supplementary file 2 — Additional file 2: Supplementary Table 2. Detailed Newcastle-Ottawa Quality Assessment Form for Cohort Studies. Supplementary Table 3. Detailed Newcastle-Ottawa Quality Assessment Form for each cross-section Studies. [file 12879_2023_8910_MOESM2_ESM.docx]

**Newcastle-Ottawa Quality Assessment Form for Cohort Studies**

Note: A study can be given a maximum of one star for each numbered item within the Selection and Outcome categories. A maximum of two stars can be given for Comparability.

**Selection**

1) Representativeness of the exposed cohort

a) Truly representative ***(one star)***

b) Somewhat representative ***(one star)***

c) Selected group

d) No description of the derivation of the cohort

2) Selection of the non-exposed cohort

a) Drawn from the same community as the exposed cohort ***(one star)***

b) Drawn from a different source

c) No description of the derivation of the non exposed cohort

3) Ascertainment of exposure

a) Secure record (e.g., surgical record) ***(one star)***

b) Structured interview ***(one star)***

c) Written self report

d) No description

e) Other

4) Demonstration that outcome of interest was not present at start of study

a) Yes ***(one star)***

b) No

**Comparability**

1) Comparability of cohorts on the basis of the design or analysis controlled for confounders

a) The study controls for age, sex and marital status ***(one star)***

b) Study controls for other factors (list) _________________________________ ***(one star)***

c) Cohorts are not comparable on the basis of the design or analysis controlled for confounders

**Outcome**

1) Assessment of outcome

a) Independent blind assessment ***(one star)***

b) Record linkage ***(one star)***

c) Self report

d) No description

e) Other

2) Was follow-up long enough for outcomes to occur

a) Yes ***(one star)***

b) No

Indicate the median duration of follow-up and a brief rationale for the assessment above:____________________

3) Adequacy of follow-up of cohorts

a) Complete follow up- all subject accounted for ***(one star)***

b) Subjects lost to follow up unlikely to introduce bias- number lost less than or equal to 20% or description of those lost suggested no different from those followed. ***(one star)***

c) Follow up rate less than 80% and no description of those lost

d) No statement

**Supplementary Table 2 Detailed Newcastle-Ottawa Quality Assessment Form for Cohort Studies**

| Studies | Selection | | | | Comparability | | Outcome | | |  |
| --- | --- | --- | --- | --- | --- | --- | --- | --- | --- | --- |
|  | Representativeness of the exposed cohort | Selection of the non-exposed cohort | Ascertainment of exposure | Demonstration that outcome of interest was not present at start of study | Comparability of cohorts on the basis of the design | analysis controlled for confounders | Assessment of outcome | Was follow-up long enough for outcomes to occur | Adequacy of follow-up of cohorts | Total quality score |
| Simeneh T. (2020) | 1 | 1 | 1 | 0 | 0 | 1 | 1 | 1 | 1 | 7 |

Note: Thresholds for converting the Newcastle-Ottawa scales to AHRQ standards (good, fair, and poor):

**Good quality:** 3 or 4 stars in selection domain AND 1 or 2 stars in comparability domain AND 2 or 3 stars in outcome/exposure domain

**Fair quality:** 2 stars in selection domain AND 1 or 2 stars in comparability domain AND 2 or 3 stars in outcome/exposure domain

**Poor quality:** 0 or 1 star in selection domain OR 0 stars in comparability domain OR 0 or 1 stars in outcome/exposure domain

**NEWCASTLE - OTTAWA QUALITY ASSESSMENT SCALE**

**(adapted for cross sectional studies)**

**Selection:** (Maximum 5 stars)

1) Representativeness of the sample:

a) Truly representative of the average in the target population. * (all subjects or random sampling)

b) Somewhat representative of the average in the target population. * (non-random sampling)

c) Selected group of users.

d) No description of the sampling strategy.

2) Sample size:

a) Justified and satisfactory. *

b) Not justified.

3) Non-respondents:

a) Comparability between respondents and non-respondents characteristics is established, and the response rate is satisfactory. *

b) The response rate is unsatisfactory, or the comparability between respondents and non-respondents is unsatisfactory.

c) No description of the response rate or the characteristics of the responders and the non-responders.

4) Ascertainment of the exposure (risk factor):

a) Validated measurement tool. **

b) Non-validated measurement tool, but the tool is available or described.*

c) No description of the measurement tool.

**Comparability:** (Maximum 2 stars)

1) The subjects in different outcome groups are comparable, based on the study design or analysis. Confounding factors are controlled.

a) The study controls for the most important factor (select one). *

b) The study control for any additional factor. *

**Outcome:** (Maximum 3 stars)

1) Assessment of the outcome:

a) Independent blind assessment. **

b) Record linkage. **

c) Self report. *

d) No description.

2) Statistical test:

a) The statistical test used to analyze the data is clearly described and appropriate, and the measurement of the association is presented, including confidence intervals and the probability level (p value). *

b) The statistical test is not appropriate, not described or incomplete.

This scale has been adapted from the Newcastle-Ottawa Quality Assessment Scale for cohort studies to perform a quality assessment of cross-sectional studies for the systematic review, “Are Healthcare Workers’ Intentions to Vaccinate Related to their Knowledge, Beliefs and Attitudes? A Systematic Review”.

We have not selected one factor that is the most important for comparability, because the variables are not the same in each study. Thus, the principal factor should be identified for each study.

In our scale, we have specifically assigned one star for self-reported outcomes, because our study measures the intention to vaccinate. Two stars are given to the studies that assess the outcome with independent blind observers or with vaccination records, because these methods measure the practice of vaccination, which is the result of true intention.

**Supplementary Table 3 Detailed Newcastle-Ottawa Quality Assessment Form for each cross-section Studies**

| Studies | Selection | | | | Comparability | | Outcome | |  |
| --- | --- | --- | --- | --- | --- | --- | --- | --- | --- |
|  | Representativeness of the sample | Sample size | Non-respondents | Ascertainment of the exposure (risk factor) | study controls for the most important factor | study control for any additional factor | Assessment of the outcome | Statistical test | Total quality score |
| Tadewos et al. (2012) | 1 | 1 | 0 | 1 | 1 | 1 | 1 | 1 | 7 |
| Kemal et al. (2020) | 1 | 1 | 0 | 1 | 1 | 0 | 1 | 1 | 6 |
| Aklog A. (2019) | 1 | 1 | 0 | 0 | 1 | 1 | 1 | 1 | 6 |
| Wondiferaw et al. (2014) | 1 | 1 | 1 | 0 | 1 | 1 | 1 | 1 | 7 |
| Tilahun et al. (2022) | 1 | 1 | 1 | 1 | 1 | 0 | 1 | 1 | 7 |
| Gebrie et al. (2020) | 1 | 1 | 0 | 1 | 1 | 1 | 1 | 1 | 7 |
| Fiseha et al. (2021) | 1 | 1 | 1 | 0 | 1 | 1 | 1 | 1 | 7 |
